# Lansoprazole-Induced Myoclonus

**DOI:** 10.5334/tohm.1236

**Published:** 2026-08-14

**Authors:** R. N. Komal Kumar, Vijay K. Sharma

**Affiliations:** 1 Department of Neurology, Yashoda Hospital, Secunderabad, Telangana, India; 2 Yong Loo Lin School of Medicine, National University of Singapore, Singapore; 3 Division of Neurology, National University Hospital, Singapore

**Keywords:** Myoclonus, lansoparazole, proton pump inhibitor

## Abstract

Although Lansoprazole, a proton pump inhibitor, is quite safe, there have been occasional reports of associated neurological dysfunction. We present a middle-aged man who developed bilateral asterixis within few hours of the first dose of lansoprazole. Interestingly, the jerks disappeared rapidly after discontinuation of lansoprazole.

## Case description

A 62-years old man presented with 2-day’s history of increasing jerking of upper limbs and head. There was no antecedent illness, fever or change in level of consciousness. He did not suffer from any chronic disease. He was having upper gastrointestinal dyspepsia for past 6-weeks for which oral lansoprazole (15 mg once-a-day) was started. The involuntary movements started about 6-hours after taking the first-dose of lansoprazole. He denied smoking, consuming alcohol or using any recreational drugs.

Upon arrival, he was afebrile, conscious, oriented and had normal speech and power in extremities. However, positive and negative myoclonus (asterixis) of upper limbs and neck was noted (Video 1). This was not related to startle or body position. Blood counts, electrolytes, drug and toxicology screen, kidney and liver function tests were unremarkable. Magnetic resonance imaging of the brain, electroencephalopgraphy (EEG) and electromyography (EMG) were planned and lansoprazole was discontinued. Since myoclonus resolved completely within 2-days of stopping lansoprazole, all planned investigations were cancelled. He remained asymptomatic when seen at outpatient clinic 2-months later.

**Video 1:** Myoclonic jerking of both upper limbs and neck muscles in a 62-years old man that started after the first dose of oral lansoprazole.

## Discussion

Lansoprazole, a proton pump inhibitor (PPI), selectively and irreversibly inhibits gastric hydrogen ion secretion. As single daily dose, it is considered slightly more effective than omeprazole for suppressing gastric acid secretion [1].

PPIs, although quite safe, may occasionally cause neurological dysfunctions. Two reported cases developed rapid onset encephalopathy, myoclonus and extrapyramidal features [2]. However, lansoprazole was co-administered with levodopa in them and its withdrawal led to the resolution of symptoms. Lansoprazole may also induce hypomagnessemia and lead to neurological disturbances [3].

Myoclonus is sudden, brief involuntary twitching, jerking, or spasm of a single muscle or a group of muscles. It can result from sudden muscle contractions (called positive myoclonus) or sudden muscle relaxation (called negative myoclonus). Myoclonus usually represents a disruption of brain or spinal cord. However, it can also happen after an injury to the peripheral nerves [4].

Myoclonus may be classified on the basis of neurophysiological findings and the site of its pathophysiologic generator [5]. Accordingly, myoclonus can be cortical, cortical-subcortical, subcortical-nonsegmental, segmental or peripheral.

Generally, asterixis occurs due to toxic or metabolic causes. Although difficult to prove, occurrence of asterixis within 6-hours of the first dose of lansoprazole and its rapid resolution after discontinuation supports our belief that this phenomenon was an idiosyncratic reaction in our patient. Since the jerking was prominently proximal, it is more likely that our patient suffered from subcortical myoclonus. We could not perform EEG (to differentiate between cortical and subcortical myoclonus) and EMG (to record the discharge duration and pattern of myoclonus) for a precise classification since the symptoms resolved rapidly after cessation of lansoprazole. Our case is a reminder that even the medications like lansoprazole, considered innocuous, may cause unexpected disabling neurological phenomenon.
